# Pneumococcal carriage and disease in adults hospitalised with community-acquired pneumonia in Mongolia: prospective pneumonia surveillance program (2019–2022)

**DOI:** 10.1186/s41479-025-00184-w

**Published:** 2025-11-25

**Authors:** Tuya Mungun, Munkhchuluun Ulziibayar, Cattram D. Nguyen, Purevsuren Batsaikhan, Bujinlkham Suuri, Dashtseren Luvsantseren, Dorj Narangerel, Bilegtsaikhan Tsolmon, Lien Anh Ha Do, Darren Suryawijaya Ong, Belinda D. Ortika, Casey L. Pell, Laura K. Boelsen, Ashleigh C. Wee-Hee, Leena Spry, Jason Hinds, Michael W. Pride, Eileen M. Dunne, Bradford D. Gessner, E. Kim Mulholland, Catherine Satzke, Claire von Mollendorf

**Affiliations:** 1 National Center for Communicable Diseases, Ministry of Health, Ulaanbaatar, Mongolia; 2 Infection, Immunity and Global Health, Murdoch Children’s Research Institute, Melbourne, Australia; 3 Department of Paediatrics, The University of Melbourne, Melbourne, Australia; 4 Ministry of Health, Ulaanbaatar, Mongolia; 5 Institute of Biomedical Sciences, Mongolian National University of Medical Sciences, Ulaanbaatar, Mongolia; 6 Institute for Infection and Immunity, St. George’s University of London, London, UK; 7 London Bioscience Innovation Centre, BUGS Bioscience, London, UK; 8 Vaccine Research and Development, Pfizer Research, Pearl River, USA; 9 Pfizer Vaccines, Collegeville, USA; 10 Department of Infectious Disease Epidemiology and International Health, School of Hygiene and Tropical Medicine, London, London, UK; 11 Department of Microbiology and Immunology, Peter Doherty Institute for Infection and Immunity, The University of Melbourne, Melbourne, Australia

**Keywords:** Pneumonia, Pneumococcal carriage, Hospitalisation, Pneumococcal conjugate vaccine, Adult, Mongolia

## Abstract

**Background:**

*Streptococcus pneumoniae* is an important cause of pneumonia in older adults, however, serotyping and indirect impact information from low and middle-income countries is lacking. Mongolia has a childhood 13-valent pneumococcal conjugate vaccine (PCV13) program, but no adult pneumococcal vaccination program. We describe pneumococcal carriage rates, disease and serotype distribution among adults hospitalised with pneumonia, and explore changes over the COVID-19 pandemic period.

**Methods:**

Adults (≥ 18 years) hospitalised with clinical pneumonia were enrolled over 3 years (March 2019-February 2022) into a prospective pneumonia surveillance program. Nasopharyngeal swabs were tested to detect pneumococci using *lytA* qPCR and molecular serotyping by DNA microarray and metagenomics. Pneumococcal pneumonia was identified using serotype-specific urinary antigen detection and BinaxNOW^®^ assays. Pneumococcal carriage and pneumonia prevalence were assessed over the COVID-19 period with log-binomial regression used to estimate prevalence and adjusted prevalence ratios (pre- versus early- and late-COVID-19 periods).

**Results:**

Of 3,178 pneumonia cases, *S. pneumoniae* was identified in 12.1% (333/2,759) of swabs and 8.6% (253/2,925) of urine samples. PCV13 serotype carriage prevalence was 3.1% (82/2,663) and non-PCV13 serotype carriage prevalence 5.7% (152/2,663). In the late-COVID-19 period, pneumococcal carriage prevalence was reduced by 66% (aPR 0.34, 95%CI 0.25–0.46) and pneumococcal pneumonia by 82% (aPR 0.18, 95%CI 0.12–0.27) compared with the pre-COVID-19 transmission period.

**Conclusion:**

Despite paediatric vaccination with high coverage, we identified some residual PCV13 serotypes with predominance of non-PCV13 serotypes carried and causing disease in adults. Direct adult vaccination which targets these serotypes will potentially reduce disease in adults in Mongolia.

**Supplementary Information:**

The online version contains supplementary material available at 10.1186/s41479-025-00184-w.

## Background

Pneumonia causes a substantial burden of disease in adults, with higher hospitalisation rates in older age groups. In 2015, it was estimated that 6.8 million hospital pneumonia admissions and 1.1 million pneumonia-related hospital deaths occurred in adults ≥ 65 years of age. Over half of these deaths were from low- and middle-income countries (LMICs) [1]. *Streptococcus pneumoniae* (the pneumococcus) accounts for around a third of adult pneumonia cases and is the leading bacterial cause worldwide [2]. However, global and country-specific pneumococcal burden is likely underestimated due to limited data on pneumococcal disease and carriage in adults with pneumonia from high burden LMICs [3].

Childhood PCV immunisation programs demonstrate indirect effects in unvaccinated adults through reductions in invasive pneumococcal disease (IPD). However, disease caused by non-vaccine serotypes and persistent vaccine-serotypes remain a concern in adults, especially in LMICs [4]. IPD data are limited in LMICs [5], particularly for adults. In these settings, pneumococcal carriage can be informative to monitor direct and indirect effects of the paediatric pneumococcal vaccine national immunisation program (NIP) [6].

It is currently unclear to what degree a paediatric PCV NIP will reduce pneumonia in unvaccinated adults in LMICs. Mongolia currently has no adult pneumococcal vaccination program. However, the country introduced childhood 13-valent pneumococcal conjugate vaccine (PCV13) in a phased strategy from 2016 [7]. Adult pneumonia surveillance was instituted in four district hospitals from 2015 to 2022 to determine indirect effects of childhood PCV13 vaccination on adult disease [8]. The study did not find evidence of indirect protection against adult all-cause and severe clinical pneumonia [9]. The highest burden of pneumonia was identified in adults ≥ 65 years [9, 10].

In response to the COVID-19 pandemic, Mongolia implemented a series of non-pharmaceutical interventions (NPIs) from the end of January 2020 [11]. These interventions and restrictions prevented local COVID-19 transmission until November 2020 [12]. NPIs have been demonstrated to interrupt viral circulation. There are few data on the impact of NPIs on pneumococcal disease and carriage in adults in LMICs. IPD data from 30 high and middle-income countries showed a reduction during the first two years of the pandemic, with increases as pandemic restrictions were lifted [13], while carriage results from high-income settings showed variable results [14, 15].

We undertook the current study to describe the distribution of pneumococcal pneumonia and carriage serotypes, to further define and explore the lack of indirect effects observed from the paediatric NIP on all-cause and severe clinical pneumonia, describe the viral causes of adult pneumonia, assess the effects of the COVID-19 pandemic on pneumococcal pneumonia and carriage epidemiology, and to help guide vaccination policy for reducing pneumonia in adults.

## Methods

### Study setting

Approximately half of Mongolia’s population (3.5 million) live in its capital city, Ulaanbaatar, with 43% of residents living in gers (traditional tents). During winter, due to temperatures frequently dropping to −40 °C and high domestic coal use, Ulaanbaatar is one of the world’s most polluted cities [16]. Pneumonia hospitalisation rates, including those for influenza and respiratory syncytial virus (RSV), are highly seasonal, with highest rates during the extremely cold winter months [17, 18].

The Mongolian Government introduced PCV13 into the childhood NIP in a phased manner by district from 2016 [7, 19]. This analysis includes data from the prospective hospital-based adult pneumonia surveillance period in four districts of Ulaanbaatar (March 2019 – February 2022) [8].

### Study population and design

As previously described, we enrolled adults with a pre-defined clinical pneumonia case definition (diagnosis of pneumonia on admission and discharge, plus two or more defined clinical signs and symptoms, of which ≥ 1 was respiratory) and admitted to one of four participating district hospitals [8]. Clinical staff completed standardised questionnaires and collected urine samples, nasopharyngeal swabs and chest x-rays (CXR).

During the COVID-19 pandemic period (from February 2020), patients with respiratory symptoms were tested for SARS-CoV-2 at time of admission using rapid antigen testing and confirmatory quantitative real-time polymerase chain reaction (qPCR). Only patients with a negative SARS-CoV-2 result were enrolled in this study and those with a positive result were transferred to a COVID-19 treatment facility [8].

### Study case definitions

As previously described [8], severe clinical pneumonia was defined as clinical pneumonia with ≥ 2 severity signs including confusion, tachypnoea, hypotension or hypoxaemia; hypoxic pneumonia as pneumonia with oxygen saturation < 90%; radiologically confirmed pneumonia as primary end-point pneumonia or any infiltrate consistent with community acquired pneumonia (CAP); pneumococcal pneumonia as clinical pneumonia with a positive BinaxNOW^®^ urinary antigen test or serotype-specific urinary antigen detection (SSUAD) assay result; and definite pneumococcal pneumonia as pneumonia with a positive blood or pleural fluid culture or BinaxNOW^®^ urinary antigen test or radiologically confirmed pneumonia with a positive SSUAD assay result [8].

### Sample collection and laboratory procedures

World Health Organization recommended methods were used for nasopharyngeal swab sample collection, handling and transport [20]. Laboratory methods have previously been described [21]. DNA was extracted from aliquoted samples followed by qPCR targeting the *lytA* gene. Molecular serotyping by DNA microarray was conducted using Senti-SPv1.5 microarrays (BUGS Bioscience) [21]. PCV13 serotypes were defined as 1, 3, 4, 5, 6A, 6B, 7F, 9V, 14, 18C, 19A, 19F and 23F. All other serotypes, including non-encapsulated pneumococci [22] were considered non-PCV13 serotypes. Serotypes 15B and 15C were reported as 15B/C [22] and 11F-like serotypes as 11A [23]. Carriage density (log_10_ genome equivalents/ml; GE/ml) was calculated by reference to a standard curve prepared from reference isolate genomic DNA [21]. The presence of ten antimicrobial resistance (AMR) genes was assessed by DNA microarray [24]. DNA from *lytA*-positive and culture-negative samples were sent for metagenomic sequencing to identify additional pneumococcal serotypes. DNA was sequenced on the Illumina NovaSeq platform. Following quality control (using fastp version 0.23.2) and removal of human reads (using Bowtie2 version 2.4.5 and Samtools Version 1.14 with the human GRCh38/hg38 release 107 reference genome), sequencing results were analysed using a standard pneumococcal serotyping tool for mixed samples (PneumoKITy [25]). Taxonomic classification of reads (using Kraken 2 version 2.1.1 with Bracken version 2.9) was used to identify pneumococcal-positive samples (Supplementary Methods). For viral testing, RNA was extracted from one aliquot and tested for RSV and influenza using a validated multiplex real time qPCR [7, 8].

Urine samples were tested using a rapid pneumococcal urinary antigen test (BinaxNOW^®^, Alere North America) [8] and Pfizer-validated multiplex SSUAD assays that detect the 13 serotypes in PCV13 and cross-reactive serotypes 6A/C, 7F/A, 9 V/9A, 18C/18A, B,F (UAD1) [26] and the additional 11 serotypes in PPSV23 and cross-reactive serotypes 10A/39, 11A/11D,F, 15B/15C, 22F/22A, 33F/33A, C, 17F/17A, 20 (20A and 20B) (UAD2) [27]. Urine testing and serotyping were performed blinded. Due to hard lockdowns in Ulaanbaatar, nasopharyngeal and urine samples could not be collected between 6 December 2020 to 28 February 2021 and 10 April to 8 May 2021. Clinical surveillance data collection continued throughout these periods.

### Statistical analysis

Data were analysed using Stata version 18.0 (College Station, TX: StataCorp LLC). Categorical variables were summarised with frequency counts and percentages. Demographic variables were summarised overall, stratified by age group and COVID-19 period. The COVID-19 period was divided into: pre-COVID-19 period prior to NPIs and restrictions (01 March 2019–31 January 2020), early-COVID-19 period which included lockdowns and NPIs but no local community spread of SARS-CoV-2 in Mongolia (01 February 2020–31 October 2020), and the late-COVID-19 period when local spread of SARS-CoV-2 occurred in Mongolia (01 November 2020–28 February 2022). Restrictions were present for part of the late period (Supplementary Methods).

Nasopharyngeal carriage and SSUAD detection rates were reported over the three COVID-19 periods. Log-binomial regression was used to estimate prevalence rates and crude and adjusted prevalence rate ratios comparing early- and late- to pre-COVID-19 period. A common set of confounders (based on relevant literature) was used to adjust prevalence ratios and included age group, sex, underlying medical conditions, housing type (formal/informal), household crowding (> 3 people per room), household fuel type, income level, season, antibiotic use in 48 h prior to admission and presence of virus. Participants with complete data for all variables in the model (complete case analysis) were included.

Carriage rates were calculated for individual PCV13 serotypes and the most common non-PCV13 serotypes. Individual carriage rates and SSUAD detection rates were plotted over the three COVID-19 periods. Pneumococcal nasopharyngeal colonisation density data were log_10_ transformed and reported as log_10_ GE/ml. Density data were compared between the three COVID-19 periods for all pneumococcal, PCV13 and non-PCV13 carriage. To determine the effect of NPIs on density, quantile regression was used to compare the median pneumococcal densities in the early- and late- to the pre-COVID-19 period. A common set of confounders (age group, underlying medical condition, previous pneumonia admission, smoking, education, income level) advised by the literature was used to adjust the regression coefficient.

Detection rates of AMR genes for all, PCV13 and non-PCV13 serotypes were determined. AMR detection rates between the three COVID-19 periods were compared. Only samples that contained a single pneumococcal serotype with no other species identified were included in the AMR analysis.

## Results

A total of 3,442 pneumonia cases were enrolled in the prospective adult pneumonia surveillance program from 01 March 2019 to 28 February 2022. Of these, 3,178 (92.3%) met the clinical pneumonia case definition and were included in the analysis. Overall, 1,177 (37.0%) patients were between 46 and 64 years old, 1,370 (43.1%) were male and 1,928 (60.8%) had one or more underlying medical conditions. The older age groups (≥ 46 years) reported more underlying medical conditions. Most cases were from Songinokhairkhan Adult Hospital and Bayanzurkh District Hospital, the two largest districts (Table 1). Some differences were observed in patient characteristics over the three COVID-19 periods (Table 2). Compared with the pre-COVID-19 period there was a higher percentage of adults ≥ 65 years (23.2% vs. 30.3%) and patients with multiple medical conditions (22.4% vs. 33.6%) in the late-COVID-19 period.

**Table 1:** Characteristics of adults hospitalised with community-acquired pneumonia included in prospective surveillance program, by age group, Ulaanbaatar, Mongolia, March 2019 − February 2022 (*N* = 3,178)

		Total	18–25 years	26–45 years	46–64 years	≥ 65 years
		*N* = 3,178 *n* (%)	*N* = 265 *n* (%)	*N* = 870 *n* (%)	*N* = 1,177 *n* (%)	*N* = 866 *n* (%)
*Demographics*						
Sex (*N* = 3,178)	Male	1,370 (43.1)	102 (38.5)	368 (42.3)	536 (45.5)	364 (42.0)
Admission hospital (*N* = 3,178)	Bayanzurkh District Hospital	906 (28.5)	88 (33.2)	274 (31.5)	337 (28.6)	207 (23.9)
	Songinokhairkhan Adult Hospital	1,149 (36.2)	88 (33.2)	306 (35.2)	440 (37.4)	315 (36.4)
	Sukhbaatar District Hospital	746 (23.5)	57 (21.5)	188 (21.6)	264 (22.4)	237 (27.4)
	Chingeltei District Hospital	377 (11.9)	32 (12.1)	102 (11.7)	136 (11.6)	107 (12.4)
Season (*N* = 3,178)	Summer	640 (20.1)	61 (23.0)	197 (22.6)	209 (17.8)	173 (20.0)
	Autumn	654 (20.6)	55 (20.8)	177 (20.3)	259 (22.0)	163 (18.8)
	Winter	1,302 (41.0)	94 (35.5)	332 (38.2)	497 (42.2)	379 (43.8)
	Spring	582 (18.3)	55 (20.8)	164 (18.9)	212 (18.0)	151 (17.4)
*Risk factors*						
Underlying medical conditions (*N* = 3,169)^†^	No medical conditions	1,241 (39.2)	186 (70.2)	519 (60.0)	373 (31.8)	163 (18.8)
	One medical condition	1,007 (31.8)	65 (24.5)	231 (26.7)	402 (34.3)	309 (35.7)
	> 1 medical condition	921 (29.0)	14 (5.3)	115 (13.3)	398 (33.9)	394 (45.5)
Household crowding (*N* = 3,159)^‡^	Yes	412 (13.0)	47 (17.9)	165 (19.1)	137 (11.7)	63 (7.3)
Antibiotics in last 48 h (*N* = 3,145)	Yes	674 (21.4)	61 (23.2)	242 (28.1)	224 (19.2)	147 (17.2)
Previous pneumonia admission (*N* = 3,125)	Yes	421 (13.5)	29 (11.2)	85 (9.8)	165 (14.2)	142 (16.8)
Previous other non-pneumonia admission (*N* = 3,106)	Yes	868 (27.9)	39 (15.1)	159 (18.6)	367 (31.9)	303 (35.9)
Any previous admission (*N* = 3,102)	Yes	1,113 (35.9)	62 (23.9)	218 (25.5)	448 (39.1)	385 (45.6)
Smoker (*N* = 3,163)	Yes	745 (23.6)	40 (15.2)	219 (25.3)	319 (27.2)	167 (19.4)
Drinks alcohol (*N* = 3,158)	Yes	395 (12.5)	9 (3.4)	129 (14.9)	185 (15.8)	72 (8.4)
*Socio-economic factors*						
Tertiary education (*N* = 3,157)	Yes	1,075 (34.1)	100 (38.2)	392 (45.3)	354 (30.3)	229 (26.6)
Informal housing (*N* = 3,166)	Yes	852 (26.9)	61 (23.2)	204 (23.5)	345 (29.4)	242 (28.0)
Smoky fuel (*N* = 3,165)	Yes	1,739 (54.9)	138 (52.3)	437 (50.4)	679 (57.9)	485 (56.3)
Below minimum income (*N* = 2,696)^§^	Yes	304 (11.3)	37 (17.2)	81 (11.4)	116 (11.5)	70 (9.2)
*Disease and severity*						
Died during admission (*N* = 3,158)	Yes	43 (1.4)	0 (0.0)	5 (0.6)	13 (1.1)	25 (2.9)
Admission duration (N = 3,178)	≤ 7 days	1,887 (59.4)	175 (66.0)	550 (63.2)	699 (59.4)	463 (53.5)
	≥ 8 days	1,291 (40.6)	90 (34.0)	320 (36.8)	478 (40.6)	403 (46.5)
Definite pneumococcal pneumonia (*N* = 2,929)^¶^	Yes	240 (8.2)	19 (7.7)	79 (9.8)	89 (8.3)	53 (6.7)
Primary end-point pneumonia (*N* = 2,332)^*^	Yes	722 (31.0)	49 (23.9)	174 (27.4)	282 (32.8)	217 (34.3)
Severe pneumonia (*N* = 3,178)^**^	Yes	322 (10.1)	16 (6.0)	64 (7.4)	125 (10.6)	117 (13.5)
Hypoxic pneumonia (*N* = 3,118)^||^	Yes	513 (16.5)	13 (5.0)	77 (9.0)	210 (18.3)	213 (25.1)
*Vaccines received in last 12 months*						
Received COVID-19 vaccine (*N* = 1,355)^#^	Yes	1,049 (77.4)	51 (77.3)	266 (78.5)	430 (79.2)	302 (73.2)
Received influenza vaccine (*N* = 3,113)^##^	Yes	40 (1.3)	2 (0.8)	14 (1.6)	15 (1.3)	9 (1.1)
*Pneumococcal carriage and urine detection*						
All pneumococcal carriage (*N* = 2,759)^^^	Yes	333 (12.1)	25 (10.9)	85 (11.2)	120 (11.9)	103 (13.6)
PCV13 serotype carriage (*N* = 2,663)^^^^	Yes	82 (3.1)	7 (3.1)	17 (2.3)	30 (3.1)	28 (3.8)
Non- PCV13 serotype carriage (*N* = 2,663)^^^^	Yes	152 (5.7)	13 (5.8)	47 (6.4)	50 (5.2)	42 (5.8)
Serotype-specific urine antigen detection assay positive (*N* = 2,925)	Yes	253 (8.6)	22 (8.9)	86 (10.7)	92 (8.5)	53 (6.7)
*Viral infections*						
Influenza infection (*N* = 2,765)	Yes	113 (4.1)	13 (5.7)	43 (5.6)	27 (2.7)	30 (3.9)
RSV infection (*N* = 2,765)	Yes	18 (0.7)	1 (0.4)	1 (0.1)	12 (1.2)	4 (0.5)

†Based on documentation in medical chart and included asthma, chronic obstructive pulmonary disease/emphysema, tuberculosis, cirrhosis/liver failure, coronary artery disease, hypertension, heart failure, chronic renal failure and diabetes. ‡Crowding considered > 3 people per room. §Minimum income was considered 216.900₮ per person/per month. ¶Definite pneumococcal pneumonia defined as any clinical pneumonia admission with a positive blood or pleural fluid culture or a positive pneumococcal urine test from BinaxNOW^®^ urinary antigen test; or any radiographically confirmed pneumonia (primary end-point pneumonia or any infiltrate consistent with CAP) with a positive multiplex SSUAD diagnostic assay result. *World Health Organization (WHO) defined primary end point pneumonia. **Severe clinical pneumonia defined as clinical pneumonia with two or more severity signs: presence of confusion (Glasgow coma scale < 15), respiratory rate ≥ 30 breaths per minute, hypotension (systolic blood pressure ≤ 90 mmHg or diastolic blood pressure ≤ 60 mmHg) or hypoxaemia (oxygen saturation < 90%). ||Hypoxic defined as an oxygen saturation < 90%. #Based on electronic vaccine records. ##Based on self-report. ^Number of all pneumococcal carriage swabs differ from PCV13 serotype and non-PCV13 serotype swabs due to exclusion of pneumococcal positive samples for which serotype was not determined. ^^ Of pneumococcal *lytA* qPCR positive swabs, 96 swabs could not be serotyped using microarray or metagenomics

**Table 2:** Characteristics of adults hospitalised with community-acquired pneumonia included in prospective surveillance program, by COVID-19 period, Ulaanbaatar, Mongolia, March 2019 − February 2022

				Pre-COVID-19 period^@^	Early-COVID-19 period^@^	Late-COVID-19 period^@^
				*N* = 965	*N* = 594	*N* = 1,619
				*n* (%)	*n* (%)	*n* (%)
*Demographics*						
Age group, years (*N* = 3,178)	18–25			120 (12.4)	59 (9.9)	86 (5.3)
	26–45			301 (31.2)	166 (27.9)	403 (24.9)
	46–64			320 (33.2)	217 (36.5)	640 (39.5)
	≥ 65			224 (23.2)	152 (25.6)	490 (30.3)
Sex (*N* = 3,178)	Male			387 (40.1)	278 (46.8)	705 (43.5)
Admission hospital (*N* = 3,178)	Bayanzurkh District Hospital			246 (25.5)	164 (27.6)	496 (30.6)
	Songinokhairkhan Adult Hospital			349 (36.2)	232 (39.1)	568 (35.1)
	Sukhbaatar District Hospital			250 (25.9)	116 (19.5)	380 (23.5)
	Chingeltei District Hospital			120 (12.4)	82 (13.8)	175 (10.8)
Season (N = 3,178)	Summer			177 (18.3)	166 (27.9)	297 (18.3)
	Autumn			143 (14.8)	159 (26.8)	352 (21.7)
	Winter			414 (42.9)	90 (15.2)	798 (49.3)
	Spring			231 (23.9)	179 (30.1)	172 (10.6)
*Risk factors*						
Underlying medical conditions (*N* = 3,169)^†^	No medical conditions			445 (46.3)	208 (35.1)	588 (36.4)
	One medical condition			301 (31.3)	221 (37.3)	485 (30.0)
	> 1 medical condition			215 (22.4)	164 (27.7)	542 (33.6)
Household crowding (*N* = 3,159)^‡^	Yes			136 (14.1)	94 (16.0)	182 (11.3)
Antibiotics in last 48 h (*N* = 3,145)	Yes			260 (27.5)	105 (17.8)	309 (19.2)
Any previous admission (*N* = 3,102)	Yes			344 (36.6)	231 (39.7)	538 (34.1)
Smoker (*N* = 3,163)	Yes			220 (22.8)	161 (27.3)	364 (22.6)
Drinks alcohol (*N* = 3,158)	Yes			107 (11.1)	107 (18.2)	181 (11.3)
*Socio-economic factors*						
Tertiary education (*N* = 3,157)	Yes			346 (36.0)	193 (32.8)	536 (33.3)
Informal housing (*N* = 3,166)	Yes			262 (27.2)	173 (29.2)	417 (25.9)
Smoky fuel (*N* = 3,165)	Yes			579 (60.1)	366 (61.8)	794 (49.3)
Below minimum income (*N* = 2,696)^§^	Yes			122 (15.5)	61 (13.0)	121 (8.4)
*Disease and severity*						
Died during admission (*N* = 3,158)	Yes			10 (1.0)	17 (2.9)	16 (1.0)
Admission duration (*N* = 3,178)	≤ 7 days			538 (55.8)	356 (59.9)	993 (61.3)
	≥ 8 days			427 (44.2)	238 (40.1)	626 (38.7)
Definite pneumococcal pneumonia (*N* = 2,929)^¶^		Yes		120 (13.0)	77 (13.3)	43 (3.0)
Primary end-point pneumonia (*N* = 2,332)^*^	Yes			229 (34.1)	172 (35.8)	321 (27.2)
Severe pneumonia (*N* = 3,178)^**^	Yes			100 (10.4)	100 (16.8)	122 (7.5)
Hypoxic pneumonia (*N* = 3,118)^||^	Yes			145 (15.4)	114 (19.3)	254 (16.0)
*Vaccines received*						
Received COVID-19 vaccine (*N* = 1,355)^#^	Yes			0 (0.0)	0 (0.0)	1,049 (77.4)
Received influenza vaccine (*N* = 3,113)^##^	Yes			11 (1.2)	4 (0.7)	25 (1.6)
*Pneumococcal carriage and urine detection*						
All pneumococcal carriage (*N* = 2,759)^^^	Yes			156 (17.9)	105 (18.5)	72 (5.5)
PCV13 serotype carriage (*N* = 2,663)^^^^	Yes			44 (5.3)	31 (5.7)	7 (0.5)
Non-PCV13 serotype carriage (*N* = 2,663)^^^^	Yes			71 (8.5)	47 (8.7)	34 (2.6)
Serotype-specific urine antigen detection assay positive (*N* = 2,925)	Yes			135 (14.6)	74 (12.8)	44 (3.1)
*Viral infections*						
Influenza infection (*N* = 2,765)	Yes			105 (12.0)	8 (1.4)	0 (0.0)
RSV infection (*N* = 2,765)	Yes			13 (1.5)	2 (0.4)	3 (0.2)

^**@**^Pre-COVID-19 period defined as March 2019-January 2020 prior to non-pharmaceutical interventions (NPIs) and restrictions, early-COVID-19 period defined as February 2020-October 2020 during period of NPIs and restrictions but no community SARS-CoV-2 transmission, and late-COVID-19 period defined as November 2020-Febrary 2022 with community SARS-CoV-2 transmission

†Based on documentation in medical chart and included asthma, chronic obstructive pulmonary disease/emphysema, tuberculosis, cirrhosis/liver failure, coronary artery disease, hypertension, heart failure, chronic renal failure and diabetes. ‡Crowding considered > 3 people per room. §Minimum income was considered 216.900₮ per person/per month

¶Definite pneumococcal pneumonia defined as any clinical pneumonia admission with a positive blood or pleural fluid culture or a positive pneumococcal urine test from BinaxNOW^®^ urinary antigen test; or any radiographically confirmed pneumonia (primary end-point pneumonia or any infiltrate consistent with CAP) with a positive multiplex SSUAD diagnostic assay result

*World Health Organization (WHO) defined primary end point pneumonia. **Severe clinical pneumonia defined as clinical pneumonia with two or more severity signs: presence of confusion (Glasgow coma scale < 15), respiratory rate ≥ 30 breaths per minute, hypotension (systolic blood pressure ≤ 90 mmHg or diastolic blood pressure ≤ 60 mmHg) or hypoxaemia (oxygen saturation < 90%). ||Hypoxic defined as an oxygen saturation < 90%

#Based on electronic vaccine records. ##Based on self-report

^Number of all pneumococcal carriage swabs differ from PCV13 serotype and non-PCV13 serotype swabs due to exclusion of pneumococcal positive samples for which serotype was not determined. ^^Of pneumococcal *lytA* qPCR positive swabs, 96 swabs could not be serotyped using microarray or metagenomics

Both smoking and alcohol use differed by sex. Overall, 23.5% of cases reported smoking, including 6.1% of females (110/1,802) and 46.7% of males (635/1,361). Overall, 12.5% of patients reported drinking alcohol regularly, including 1.7% (30/1,802) of females and 26.9% (365/1,356) of males. Approximately a third of cases (26.9%) lived in informal (traditional ger) housing and 55% were exposed to smoky fuels in the home.

Among pneumonia cases, 1.4% died during admission, 8.2% had pneumococcal pneumonia, 31.0% had consolidation on CXR, 10.1% had severe pneumonia, and 16.5% had hypoxic pneumonia. These disease severity measures were generally highest in the oldest age group (Table 1). All 43 patients that died met the case definition for severe pneumonia. Patients with severe pneumonia were admitted for slightly longer (8 days, interquartile range [IQR] 7–10) than those with non-severe pneumonia (7 days, IQR 7–8, *p* < 0.001).

Influenza vaccination in the year before hospital admission was reported by 1.3% of patients. During the period of COVID-19 vaccine availability from February 2021, 77.4% of patients received one or more doses. The most common COVID-19 vaccine administered as part of the primary two dose schedule was Sinopharm/BBIBP-CorV (884/1,049, 84.2%), while the most common booster vaccine was Pfizer-BioNTech (179/230, 77.8%).

Of the 2,765 swabs tested for viruses, 130 (4.7%) samples tested positive for any virus with 113 (4.1%) positive for influenza (influenza A *n* = 81 and influenza B *n* = 32, no co-detections). The highest influenza detection rates were in December 2019 (22/95, 23.2%) and January 2020 (74/223, 33.2%). No influenza cases were detected from March 2020 to February 2022 (Supplementary Figure S1). If this period was excluded, the average influenza detection rate was 11.7% from March 2019 to February 2020. Eighteen (0.7%) samples were positive for RSV (RSV A *n* = 10, RSV B *n* = 4, both RSV A&B *n* = 4) with 14 in the pre-COVID-19 period. Only one sample was positive for both RSV and influenza (Table 1).

A total of 2,759 swabs were tested for pneumococcal carriage. Of these, 333 (12.1%) were *lytA* positive, with 144 non-culturable. A serotype was identified in 228 (68.5%) samples, including 180 by microarray and 48 by metagenomic sequencing. PCV13 serotype carriage prevalence was 3.1% (*n* = 82) (most commonly serotype 3) while non-PCV13 serotype carriage prevalence was 5.7% (*n* = 152) (most commonly serotypes 8, 11A, 15A and 34). Of all pneumococcal carriers, PCV13 serotypes accounted for 24.6%, PCV15 serotypes 25.2%, PCV20 serotypes 36.9%, PCV21 serotypes 41.7% and PPSV23 serotypes 37.8%. In 2019 some PCV13 carriage with a predominance of non-PCV13 carriage was observed; the most common serotypes were 3, 8, 11A, and 15A. By the end of the study (2021/2022), 15A, 34 and 8 were the most common serotypes carried (Supplementary Figure S2).

A total of 2,925 urines were tested using the rapid BinaxNOW^®^ test and for the 24 pneumococcal serotypes in UAD1 and UAD2. Overall, 140 (4.8%) urines tested BinaxNOW^®^ positive and SSUAD was positive in 253 (8.6%); PCV13 serotypes were identified in 167 (5.7%) samples (most commonly serotypes 3, 7F, 1), PCV15-nonPCV13 serotypes in 2 (0.1%), and PCV20-nonPCV13 serotypes in 42 (1.6%) (most commonly 8 and 10A), and PPSV23 unique serotypes in 26 (0.8%). Non-PCV20 serotypes were identified in 12 (0.4%) samples, most commonly 9N. In 2019 and 2020 the most common serotypes were 3, 8, 7F and 1. By 2021/2022 absolute counts of these serotypes were all reduced, but among the smaller number of any pneumococcal positivity, 8, 3 and 7F were still amongst the most common serotypes identified together with 6A (Supplementary Figure S2).

**Fig. 1:**
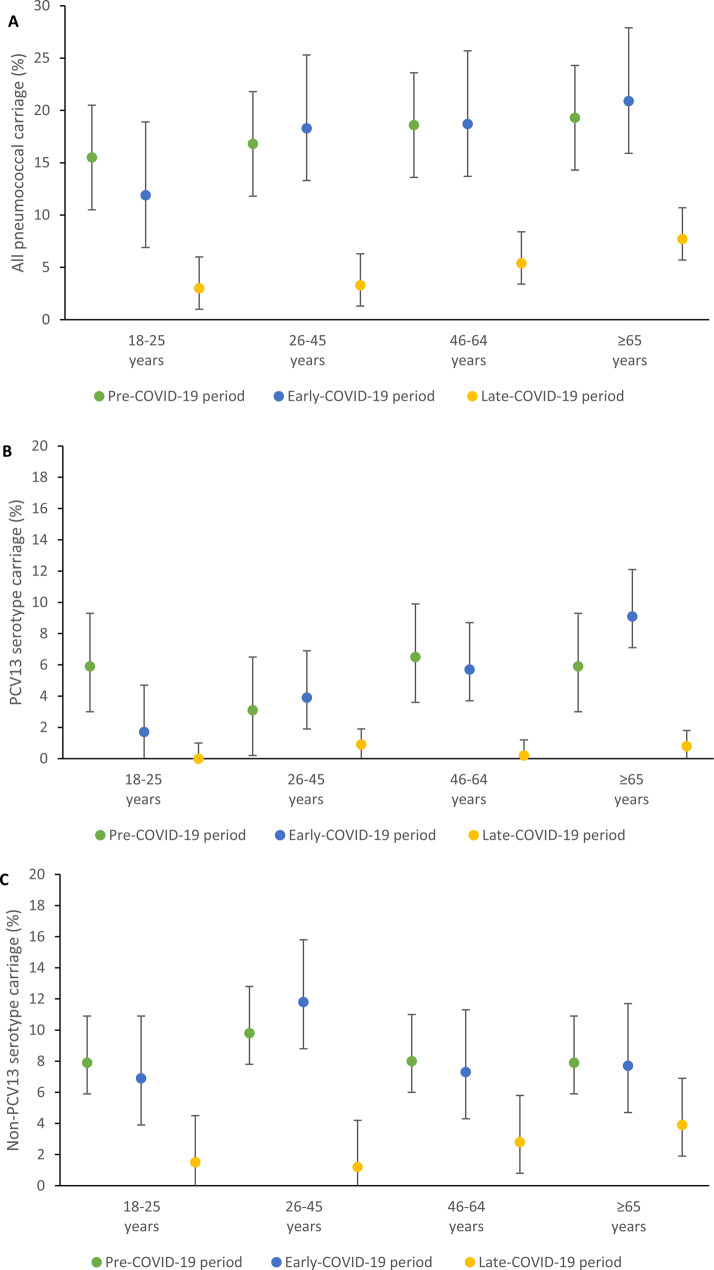
Nasopharyngeal carriage before and during the COVID-19 period by age group in adults ≥ 18 years, Mongolia, March 2019 to February 2022: (**A**) All pneumococcal carriage, (**B**) PCV13 serotype carriage, (**C**) Non-PCV13 serotype carriage. Pre-COVID-19 period (01 March 2019–31 January 2020); Early-COVID-19 period (01 February 2020–31 October 2020) which included period of lockdowns and non-pharmaceutical interventions but no local spread of SARS-CoV-2 in Mongolia, and Late-COVID-19 period (01 November 2020–28 February 2022) when local spread of SARS-CoV-2 occurred in Mongolia. Restrictions were also present for some of this period

**Fig. 2:**
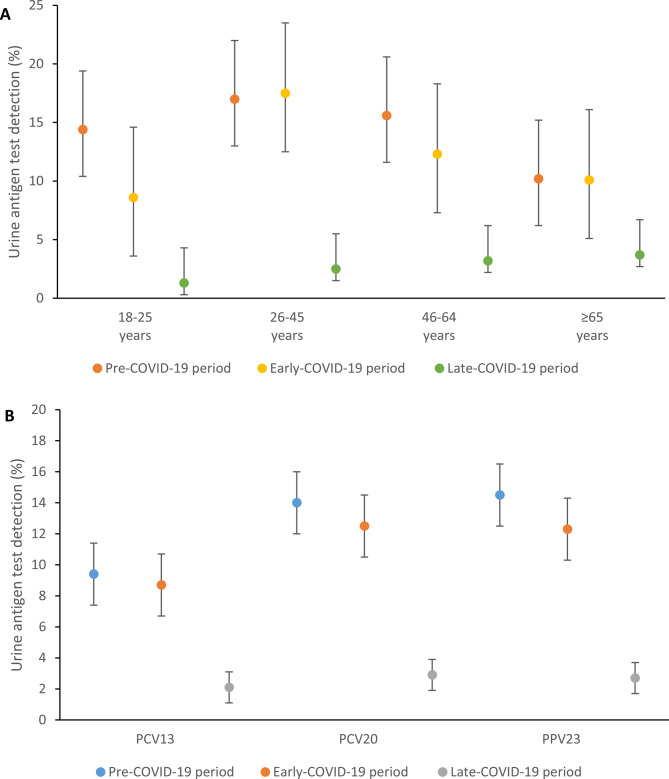
Pneumococcal serotype-specific urine antigen detection before and during the COVID-19 period by (A) Age group, (B) Vaccine type, in adults ≥ 18 years, Mongolia, March 2019 to February 2022. Pre-COVID-19 period (01 March 2019–31 January 2020); Early-COVID-19 period (01 February 2020–31 October 2020) which included period of lockdowns and non-pharmaceutical interventions but no local spread of SARS-CoV-2 in Mongolia, and Late-COVID-19 period (01 November 2020–28 February 2022) when local spread of SARS-CoV-2 occurred in Mongolia. Restrictions were also present for some of this period

**Fig. 3:**
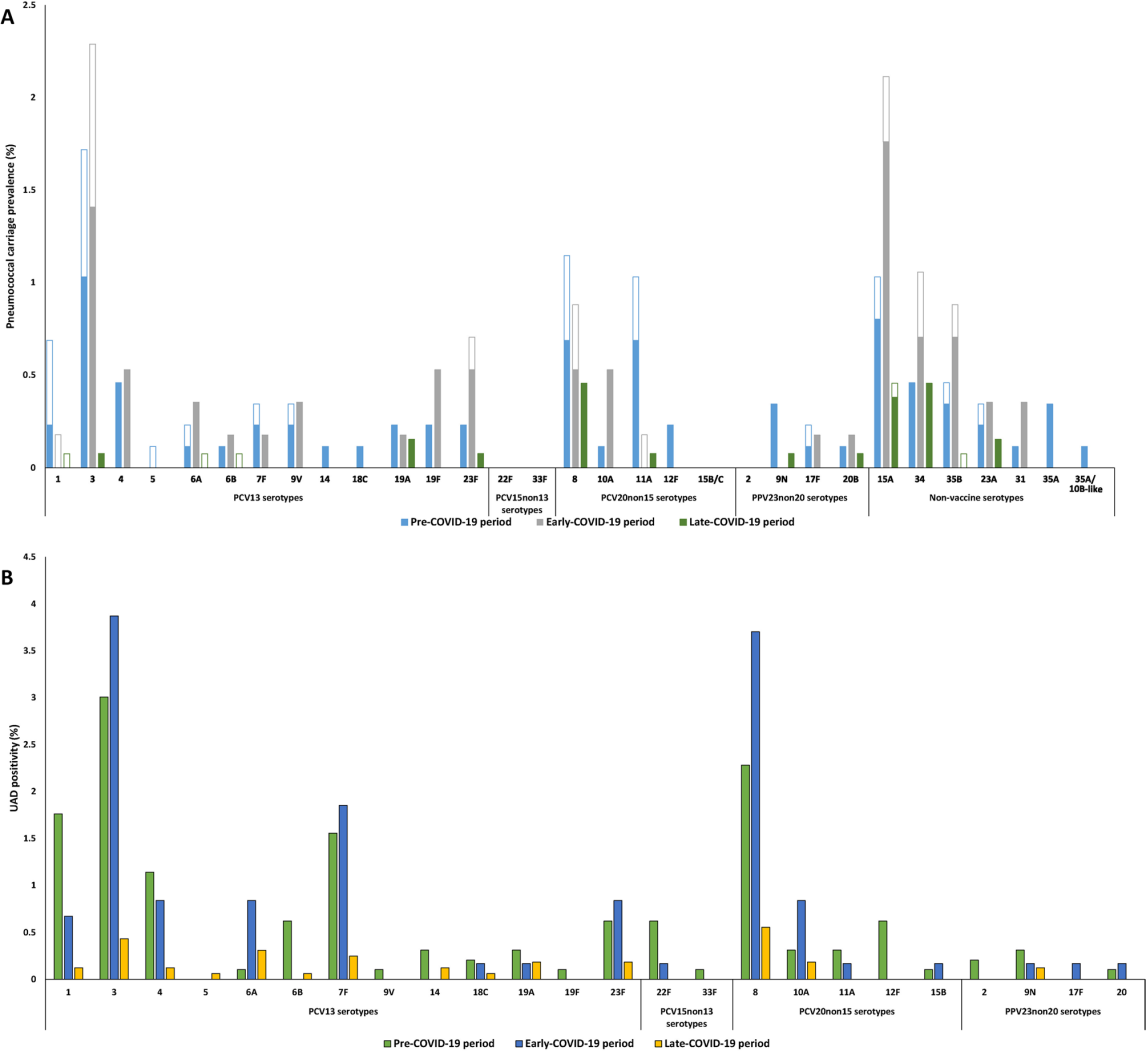
Individual serotypes identified before and during the early- and late-COVID-19 period in adults ≥ 18 years in Mongolia, between March 2019 to February 2022 **A**. PCV13 and most common non-PCV13 serotype nasopharyngeal carriage. Solid bars indicate carriage that was detected as a single or major (dominant) serotype, open bars indicate carriage that was detected using metagenomics or as a minor (second) serotype. Serotype designated ‘35A/10B-like’ on microarray identified to be new serotypes 33G (*n* = 1) [39] and 33H (*n* = 2) [40] on further investigation **B**. Serotype specific urine antigen detection (SSUAD) positive prevalence

In the late- versus pre-COVID-19 period, pneumococcal carriage (5.5% vs. 17.9%), SSUAD positivity (3.1% vs. 14.6%) and viral infections were reduced (0.2% vs. 13.5%) (Table 2). There was a substantial reduction in prevalence for all pneumococcal carriage (adjusted prevalence ratios [aPR] 0.34, 95% CI 0.25–0.46), PCV13 carriage (aPR 0.12, 95% CI 0.05–0.27), non-PCV13 carriage (aPR 0.29, 95% CI 0.19–0.47), and all UAD positivity (aPR 0.18, 95% CI 0.12–0.27) in the late- compared with pre-COVID-19 period (Supplementary Table S1). Reduced pneumococcal carriage and disease in the late-COVID-19 period was observed across all age groups and PCV13 serotypes (Figs. 1 and 2). Fig. 3A shows the carriage prevalence of all PCV13 serotypes and the most common non-PCV13 serotypes across the three defined COVID-19 periods (3, 15A, 34, 8) and Fig. 3B the most common individual serotypes detected by SSUAD (3, 8, 7F).

No difference in all or vaccine-specific (PCV13 and non-PCV13) pneumococcal nasopharyngeal carriage density was observed across the three COVID-19 periods. However, numbers were small especially for PCV13 serotype density analyses (Supplementary Table S2). The median density of PCV13 and non-PCV13 serotypes were similar overall (5.78, 95% CI 5.05–6.77 vs. 5.88, 95% CI 5.19-6.46) and at each timepoint (Supplementary Table S2 and Figure S3). There was no significant difference in detection of any or multiple AMR genes across the three COVID-19 periods. There was some variability in detection of individual AMR genes across these periods, but there was no consistent trend in these changes (Supplementary Table S3). AMR genes did not vary across PCV13 and non-PCV13 serotypes (Supplementary Table S4).

Of the 2,687 patients who had both nasopharyngeal and urine samples, 114 had pneumococcus detected in both sample types; serotyping results were available for 102 of these nasopharyngeal samples (including results from microarray and metagenomic testing). Where the same serotype was found in the paired samples, serotypes 3 (*n* = 20), 8 (*n* = 16), 1 (*n* = 8) and 4 (*n* = 6) were most common (Supplementary Table S5). The concordance for serotypes that could be detected by both NPS and UAD testing methods was 79% and discordant results were usually detected in patients with more than one serotype identified on carriage or UAD (Supplementary Table S5).

## Discussion

There are minimal data on adult pneumococcal carriage and disease from LMICs, which reduces the ability to assess the potential benefit and impact of public health interventions such as direct pneumococcal vaccination. In this study, we identified the most common pneumococcal serotypes carried and causing disease in adults hospitalised with pneumonia in Ulaanbaatar, Mongolia, in the years following implementation of a childhood PCV13 NIP. Over the three-year prospective surveillance period (March 2019 to February 2022) we found persistence of carriage and disease due to some PCV13 serotypes (serotypes 3, 7F and 1) and emergence of non-PCV13 serotypes (serotypes 8 and 15A). These changes were in the context of no demonstrated indirect effect of childhood PCV13 vaccination on adult all-cause pneumonia, but alongside disruption by the COVID-19 pandemic. Our results have implications for adult pneumococcal vaccine policy as high burden countries may need to consider protection for vulnerable adult groups, including the elderly and those with underlying medical conditions.

A systematic review and meta-analysis on the proportion of non-invasive pneumococcal CAP due to vaccine-serotypes and all serotypes in adults age ≥ 50 years only identified studies from high income countries [3]. The estimated proportion of pneumococcal CAP was influenced by the detection method, ranging from 14% using sputum/blood culture alone, 17% using a 14-valent SSUAD and 30% using a 24-valent SSUAD, and was likely underestimated. Approximately 49% of serotypes were covered by PCV13, although this varied (37% to 66%) according to testing method used [3]. Only one previous study which compared carriage and disease-causing serotypes in adults hospitalised with pneumonia was identified. This study was from the UK and demonstrated good concordance between serotypes identified from nasopharyngeal swabs and urine [28].

Very few studies from LMICs in the Asian region have reported on pneumococcal serotypes causing non-invasive pneumonia in adults; available studies for adults are usually for IPD, of limited duration or have small case numbers [29]. In our study, definite pneumococcal pneumonia was diagnosed in 8.2% of participants, with 55% of serotypes contained in PCV13; however, our definition was restricted to SSUAD positive patients with positive CXRs which would underestimate total burden. In addition, PCR testing was not used on blood or pleural fluid, and sputum collection and culture was not performed. The COVID-19 pandemic also likely contributed to the lower percentage of pneumococcal CAP in our study compared with other adult studies. This reduction was observed in other pneumococcal pneumonia studies over the COVID-19 period [30].

Persistence of certain PCV13 serotypes have been reported from several countries with well-established childhood PCV programs [3]. In the UK, persistence of PCV13 serotypes (e.g. 19F and 3) causing IPD in adults was noted with an increase in non-PCV13 serotypes (e.g. serotype 8) [31]. In South Africa, in adults aged 25 + years, although there was a significant reduction in vaccine-serotypes, some IPD was still caused by serotypes 4, 19F, 19A and 3, with a significant increase in non-PCV13 serotype IPD, especially serotypes 8 and 12 F [32]. In Malawi significant reductions in PCV13 serotype IPD were not observed in adults despite high uptake of PCV13 in children [4]. We identified several PCV13 serotypes, including 3, 7F and 1, that were carried or detected in adults despite high PCV coverage (~ 96%) in children in Mongolia, indicating the limits of relying on a paediatric NIP to indirectly protect unvaccinated adults in this setting and in contrast to what has been observed for IPD in many settings [33]. It highlights the potential increased utility of direct adult vaccination in Mongolia and potentially other high transmission settings. Carriage of non-PCV13 serotypes such as 15A, 34 and 8, was also high. Several adult pneumococcal vaccines may be useful in reducing pneumonia with variable coverage of disease (by SSUAD: 5.7% PCV13 serotypes, 6.0% PCV15 serotypes, 8.5% PCV20 serotypes) in this setting. While we could not assess disease coverage for the PCV21 vaccine as not all relevant serotypes were included in the SSUAD assays used, 39.1% of all pneumococci carried by adults in our study are included in the adult PCV21 vaccine [34].

During the COVID-19 pandemic several countries implemented NPIs to control the spread of SARS-CoV-2. Several studies have assessed the effects of these NPIs on different diseases and outcomes. A 53% reduction in IPD was reported from a network of microbiological laboratories across 30 countries [13]. A significant reduction was noted in the case count of all age groups and major serotypes in 2020–2021, however, IPD cases began to increase late in some countries in 2021 as pandemic restrictions were relaxed [13]. Our study demonstrated a substantial 82% reduction in UAD positivity up to February 2022. As COVID-19 vaccination coverage increased in Mongolia in 2021, restrictions were gradually decreased with a resultant increase in COVID-19 cases [35, 36].

Although several studies have explored pneumococcal carriage in children during the pandemic and found no reduction, data on the impact of NPIs on adult pneumococcal carriage are more limited. One study from Denmark in 1,556 adults ≥ 64 years of age (January 2019 - December 2021), identified a reduction in the carriage rate during the lockdown period (from 12.9% pre-pandemic to 4.2%) and a subsequent return to prior levels when lockdown measures were lifted [14]. A study from the United States on older adults who had regular contact with children showed consistent levels of pneumococcal carriage despite some NPIs [15]. We demonstrated a 66% sustained reduction in nasopharyngeal carriage in the late-COVID-19 period. It is possible that the extensive mitigations, lack of viral circulation and reduced contact with children in our cohort during the COVID-19 period contributed to this reduction in adult carriage.

We identified very few cases of influenza and RSV during the study. Viral infections are highly seasonal in Mongolia, with case numbers peaking during the cold winter season. Influenza vaccination coverage was very low in our cohort, likely reflecting that annual autumn influenza vaccination campaigns target high risk groups and prioritise young children. When the Mongolian Government instituted COVID-19 mitigation measures towards the end of January 2020 [11] this likely interrupted the 2019/2020 influenza season, with ongoing NPI use disrupting subsequent influenza seasons. NPIs have been demonstrated to interrupt the circulation of viruses such as influenza and RSV, with this decrease in viruses partly responsible for reduced IPD prevalence [37]. The highest number of pneumococcal cases in our study were detected in the same months as viral cases and a more comprehensive approach to adult vaccination in Mongolia including influenza vaccine and PCV may help to reduce this dual burden. Too few RSV cases were identified to comment on the use of this vaccine in adults in Mongolia.

Our study had several strengths. We prospectively and systematically collected data and samples over three years, and it is the largest adult pneumonia surveillance program with urine and nasopharyngeal carriage samples in a LMIC. Testing was performed using validated methods for disease [26, 27] and sensitive molecular methods for pneumococcal carriage and serotyping [24]. Testing methods for carriage and serotyping were consistent with those used for the paediatric pneumonia surveillance program [38]. In terms of limitations, although the COVID-19 pandemic allowed us to assess the impact of NPIs on pneumococcal carriage and disease, it created other pressures on the healthcare system, for example that specimen taking was halted during lockdowns. To mitigate these issues, we only included participants with specimens in our analysis, excluded patients with a positive SARS-CoV-2 result, and described our data according to three distinct COVID-19 pandemic periods to account for some of these changes. In addition, pneumococcal serotype data were only available in the post-PCV introduction period. We were therefore not able to show the indirect impact of childhood PCV on pneumococcal carriage and disease in adults. However, no indirect impact, was observed on adult all-cause pneumonia in Mongolia over a seven year period [9]. All participants were hospitalised with pneumonia and no healthy controls were available to compare carriage rates and serotype distribution. Lastly, the study was conducted in four districts of the capital city and so findings may not be translatable to the rural areas of Mongolia. However, findings may be relevant in similar urban settings in other countries.

## Conclusions

This study has public health policy implications by providing data on the pneumococcal pneumonia burden and relevant serotypes in an adult population in a high-burden LMIC. In addition, our findings also highlight the potential role that viral circulation has on pneumococcal epidemiology. Although carriage and disease rates were low, likely due to the COVID-19 pandemic, PCV13 serotypes were still carried by a quarter of carriers. We found that higher valency PCVs would cover 24–39% of the pneumococcal serotypes we detected. Direct adult pneumococcal vaccination will potentially reduce pneumonia in this age group, especially in conjunction with the implementation of other interventions (such as influenza vaccination and improved air quality) to reduce disease burden.

## Supplementary Information

Supplementary Material 1.

## Data Availability

All relevant data are within the paper and the Supplementary information files.
