# Supplementary material for: Pneumococcal carriage and disease in adults hospitalised with community-acquired pneumonia in Mongolia: prospective pneumonia surveillance program (2019–2022)
Source: Pneumonia (Nathan). 2025 Nov 25;17:27. doi: 10.1186/s41479-025-00184-w (PMC12645769; doi:10.1186/s41479-025-00184-w)
Supplement: Supplementary file 1 — Supplementary Material 1. [file 41479_2025_184_MOESM1_ESM.pdf]

Supplementary material for "Pneumococcal Carriage and Disease in Adults Hospitalised with Community-Acquired Pneumonia in Mongolia: prospective pneumonia surveillance program (2019-2022)"

1. Supplementary methods 2

2. Supplementary results 2

3. Supplementary Tables 4

4. Supplementary Figures 9

5. References 12

1. Supplementary Methods

For metagenomic sequencing DNA was extracted from the Skim milk-Tryptone-Glucose-Glycerol (STGG) swab media[1] and sequenced on the Illumina NovaSeq platform to an approximate depth of 8 million reads per sample. Quality control was done using fastp version 0.23.2[2] with removal of human reads using Bowtie2 version 2.4.5[3] and Samtools Version 1.14[4] with the human GRCh38/hg38 release 107 reference genome.[5] We analysed sequencing data using a standard pneumococcal serotyping tool for mixed samples (PneumoKITy) with adjusted parameters to account for the lower proportion of pneumococcal sequencing data in the samples.[6] Taxonomic classification of reads, using Kraken 2 version 2.1.1[7] with Bracken version 2.9,[8] was used to identify pneumococcal-positive samples.

In response to the COVID-19 pandemic, Mongolia implemented a series of non-pharmaceutical interventions (NPIs) from the end of January 2020, which included border controls, travel restrictions, quarantine, school closures, use of face masks, and public health measures.[9] The COVID-19 period was divided into the pre-COVID-19 period prior to NPIs and restrictions (01 March 2019 – 31 January 2020), early-COVID-19 period with lockdowns and NPIs but no local community spread of SARS-CoV-2 in Mongolia (01 February 2020 – 31 October 2020), and late-COVID-19 period with local spread of SARS-CoV-2 occurred in Mongolia (01 November 2020 – 28 February 2022). Restrictions including lockdowns were also present for some of this last period.[10]

2. Supplementary Results

A total of 2,759 nasopharyngeal swabs were tested for pneumococcal carriage; 333 (12.1%) were *lytA* positive and 189 of these could be cultured. All 144 nasopharyngeal samples that were *lytA*-positive and culture-negative were sent for metagenomic sequencing. Sequencing was completed on 142 samples (n=2 samples had very low DNA concentrations and were unable to be sequenced). We confirmed pneumococci were present in 116 (81·7%), and we were able to detect a serotype in 50 (43·1%), of sequenced samples. For two samples, the resolution between potential serotypes was too low and these were deemed 'not determined'.

For 11 samples, metagenomics could not distinguish between pairs of serotypes. Seven were both non-PCV13 serotypes and were included in the relevant non-PCV13 serotype group analyses. Four samples included a PCV13 and non-PCV13 serotype (6A/6C, 6B/6E, 7A/7F, 9A/9V), and were assigned as a PCV13 serotype as the relevant non-PCV13 serotype was not identified in another sample in the database. Overall, we obtained serotyping results for 228 nasopharyngeal samples, 180 by microarray and 48 by metagenomics.

A higher proportion of samples were non-culturable in the late-COVID-19 period (41/72, 56·9%) compared with the pre-COVID-19 (63/156, 40·4%) and early-COVID-19 periods (40/105, 38·1%). In addition, fewer non-culturable samples had a serotype determined by metagenomics in the late-COVID-19 period (9/41, 21·9%) compared with the pre-COVID-19 (24/63, 38·1%) and early-COVID-19 periods (15/40, 37·5%).

3. Supplementary Tables

Supplementary Table S1. Pneumococcal carriage (all, PCV13 serotypes, and non-PCV13 serotypes) and urine antigen detection prevalence and prevalence ratios in pre-, early- and late-COVID-19 periods.

	Number of cases (n/N)	Prevalence (%) (95% CI)	Unadjusted prevalence ratio (95% CI)	Adjusted prevalence ratio[a] (95% CI)
**All pneumococcal carriage[b]**				
Pre-COVID-19 period[c]	156/873	17·9 (15·4 - 20·6)	Reference	Reference
Early-COVID-19 period[c]	105/568	18·5 (15·4 - 21·9)	1·03 (0·83 - 1·29)	1·11 (0·85 - 1·44)
Late-COVID-19 period[c]	72/1,318	5·5 (4·3 - 6·8)	0·30 (0·23 - 0·40)	0·34 (0·25 - 0·46)
**PCV13 serotype carriage**				
Pre-COVID-19 period	44/834	5·3 (3·9 - 7·0)	Reference	Reference
Early-COVID-19 period	31/543	5·7 (3·9 - 8·0)	1·08 (0·69 - 1·69)	1·13 (0·65 - 1·96)
Late-COVID-19 period	7/1,286	0·5 (0·2 - 1·1)	0·10 (0·05 - 0·23)	0·12 (0·05 - 0·27)
**Non-PCV13 serotype carriage**				
Pre-COVID-19 period	71/834	8·5 (6·7 - 10·6)	Reference	Reference
Early-COVID-19 period	47/543	8·7 (6·4 - 11·3)	1·02 (0·71 - 1·45)	1·09 (0·72 - 1·64)
Late-COVID-19 period	34/1,286	2·6 (1·8 - 3·7)	0·31 (0·21 - 0·46)	0·29 (0·19 - 0·47)
**Serotype-specific urine antigen detection assay positive**				
Pre-COVID-19 period	135/922	14·6 (12·4 - 17·1)	Reference	Reference
Early-COVID-19 period	74/578	12·8 (10·2 - 15·8)	0·87 (0·67 - 1·14)	0·83 (0·61 - 1·13)
Late-COVID-19 period	44/1,425	3·1 (2·2 - 4·1)	0·21 (0·15 - 0·29)	0·18 (0·12 - 0·27)
**Serotype-specific urine antigen detection assay 1 (UAD1) positive**				
Pre-COVID-19 period	89/922	9·6 (7·8 - 11·7)	Reference	Reference
Early-COVID-19 period	50/578	8·6 (6·5 - 11·2)	0·90 (0·64 - 1·25)	0·89 (0·61 - 1·29)
Late-COVID-19 period	31/1,425	2·2 (1·5 - 3·1)	0·22 (0·15 - 0·34)	0·19 (0·11 - 0·31)
**Serotype-specific urine antigen detection assay 2 (UAD2) positive**				
Pre-COVID-19 period	48/922	5·2 (3·9 - 6·8)	Reference	Reference
Early-COVID-19 period	30/578	5·2 (3·5 - 7·3)	1·00 (0·64 - 1·55)	0·94 (0·53 - 1·66)
Late-COVID-19 period	14/1,425	1·0 (0·5 - 1·6)	0·19 (0·10 - 0·34)	0·18 (0·08 - 0·38)

[a] The following variables were used to adjust the prevalence ratios in each group: age group, sex, underlying medical conditions, housing type (formal or informal), household crowding (greater than three people per room), household fuel type, income level, season, antibiotic use in last 48 hours and presence of virus.

[b] All carriage prevalence does not necessarily equal the sum of PCV13 serotype and non-PCV13 serotype prevalence. This is due to multiple serotype carriage and/or exclusion of pneumococcal-positive samples for which serotype was not determined.

[C] Pre-COVID-19 period, 01 March 2019 – 31 January 2020; Early-COVID-19 period, 01 February 2020 – 31 October 2020, which included lockdown periods and non-pharmaceutical interventions but no local spread of SARS-CoV-2 in Mongolia; Late-COVID-19 period, 01 November 2020 – 28 February 2022, when local community spread of SARS-CoV-2 occurred in Mongolia. Restrictions were also present for some of this period.

Supplementary Table S2: Median density and quantile regression analysis of all pneumococci, PCV13 serotypes and non-PCV13 serotypes in pre-COVID-19, early COVID-19 and late COVID-19 periods in adults with clinical pneumonia who were pneumococcal carriers.

	N§	Median density (IQR)*	Unadjusted coefficient (95% CI)†	P value	Adjusted coefficient (95% CI)‡	P value
**All pneumococci**	333	5·46 (4·58 - 6·27)				
Pre-COVID-19 period	156	5·39 (4·62 - 6·21)	Reference		Reference	
Early-COVID-19 period	105	5·72 (4·59 - 6·44)	0·27 (-0·12 - 0·66)	0·17	0·32 (-0·18 - 0·82)	0·21
Late-COVID-19 period	72	5·34 (4·28 - 6·09)	-0·14 (-0·58 - 0·30)	0·54	-0·08 (-0·63 - 0·47)	0·77
**PCV13 serotypes****	82	5·78 (5·05 - 6·77)				
Pre-COVID-19 period	44	5·76 (4·84 - 7·00)	Reference		Reference	
Early-COVID-19 period	31	5·79 (5·06 - 6·77)	0·01 (-0·82 - 0·85)	0·97	0·10 (-0·75 - 0·96)	0·81
Late-COVID-19 period	7	5·87 (5·31 - 6·32)	0·10 (-1·35 - 1·56)	0·89	0·17 (-1·10 - 1·45)	0·78
**Non-PCV13 serotypes****	152	5·88 (5·19 - 6·46)				
Pre-COVID-19 period	71	5·78 (5·06 - 6·42)	Reference		Reference	
Early-COVID-19 period	47	6·03 (5·30 - 6·46)	0·25 (-0·24 - 0·74)	0·31	0·28 (-0·22 - 0·79)	0·27
Late-COVID-19 period	34	5·97 (5·38 - 6·65)	0·18 (-0·36 - 0·73)	0·51	0·22 (-0·37 - 0·82)	0·46

§Number of pneumococcal carriers. *Density reported in log_{10} genome equivalents/ml and interquartile range (IQR). †Coefficient is the difference in medians as determined by quantile regression, reported with 95% confidence intervals (CI). The early-and late-COVID-19 periods were compared separately with the pre-COVID-19 period. ‡Adjusted for age group, underlying medical condition, previous pneumonia admission, smoking, education and household income level.

**Serotypes identified by metagenomics were included in the density analysis.

Supplementary Table S3: Antimicrobial resistance (AMR) genes detected by microarray in nasopharyngeal samples from adults hospitalised with clinical pneumonia, shown in pre-COVID-19, early COVID-19 and late COVID-19 periods.*

AMR gene	Pre-COVID-19 period (N = 71) n (%)	Early-COVID-19 period (N = 47) n (%)	Late-COVID-19 period (N = 28) n (%)	p-value†
tetM	38 (53·5)	33 (70·2)	13 (46·4)	0·08
tetK	11 (15·5)	5 (10·6)	2 (7·1)	0·48
tetO	0 (0·0)	0 (0·0)	0 (0·0)	N/A
tetL	1 (1·4)	0 (0·0)	0 (0·0)	0·59
cat	18 (25·3)	15 (31·9)	10 (35·7)	0·54
mefA	10 (14·1)	10 (21·3)	7 (25·0)	0·38
aphA3	4 (5·6)	1 (2·1)	4 (14·3)	0·10
sat4	4 (5·6)	1 (2·1)	4 (14·3)	0·10
ermB	25 (35·2)	25 (53·2)	7 (25·0)	0·03
ermC	15 (21·1)	6 (12·8)	10 (35·7)	0·06
Any antimicrobial resistance gene	54 (76·1)	36 (76·6)	21 (75·0)	0·99
Multiple antimicrobial resistance genes**	18 (25·3)	16 (34·0)	8 (28·6)	0·59

*Only samples that contained a single pneumococcal serotype with no other species identified by microarray were included in the analysis.

†p-values compared the three COVID-19 defined periods using chi-squared test.

**≥3 antimicrobial resistance genes detected.

Supplementary Table S4: Antimicrobial resistance (AMR) genes detected by microarray in nasopharyngeal samples from adults hospitalised with clinical pneumonia.* Detection rate of antimicrobial resistance genes shown for all pneumococci, PCV13 and non-PCV13 serotypes.

AMR gene	Encodes resistance to	Overall pneumococci (N = 140) n (%)	PCV13 serotypes (N = 45) n (%)	Non-PCV13 serotypes (N = 85) n (%)	p-value†
tetM	tetracycline	84 (60·0)	26 (57·8)	57 (67·1)	0.29
tetK	tetracycline	17 (12·1)	8 (17·8)	8 (9·4)	0.17
tetO	tetracycline	0 (0·0)	0 (0·0)	0 (0·0)	N/A
tetL	tetracycline	1 (0·7)	0 (0·0)	0 (0·0)	N/A
cat	chloramphenicol	41 (29·3)	14 (31·1)	24 (28·2)	0.73
mefA	macrolides	27 (19·3)	12 (26·7)	15 (17·6)	0.23
aphA3	kanamycin	9 (6·4)	2 (4·4)	5 (5·9)	0.73
sat4	streptothricin	9 (6·4)	2 (4·4)	5 (5·9)	0.73
ermB	erythromycin	57 (40·7)	17 (37·8)	40 (47·1)	0.31
ermC	erythromycin	29 (20·7)	9 (20·0)	17 (20·0)	1.00
Any antimicrobial resistance gene		109 (77.9)	33 (73·3)	70 (82·3)	0·23
Multiple antimicrobial resistance genes**		41 (29.3)	16 (35·6)	23 (27·1)	0·31

*Only samples that contained a single pneumococcal serotype with no other species identified by microarray were included in the analysis.

†p-values compared VT versus NVT serotypes using chi-squared test.

**≥3 antimicrobial resistance genes detected.

Supplementary Table S5. Serotypes detected in paired samples on serotype specific urine antigen detection assays (UAD-1 and UAD2) and nasopharyngeal swabs (NPS) in adults ≥18 years with clinical pneumonia in Mongolia, 2019-2022.

	Serotypes detected in urine (UAD)																				
**Serotypes detected in NPS**	**1**	**10A**	**11A**	**12F**	**14**	**17F**	**18C**	**19A**	**19F**	**20**	**22F**	**23F**	**3**	**4**	**6A**	**6B**	**7F**	**8**	**9N**	**9V**	**Total**
**1**	8*	0	0	0	0	0	0	0	0	0	0	1^	0	0	0	0	0	0	0	0	**9**
**11A**	0	0	2	0	0	0	0	0	0	0	0	0	0	1**	0	0	0	1	0	0	**4**
**12F**	0	0	0	1	0	0	0	0	0	0	0	0	0	0	0	0	0	0	0	0	**1**
**13**	0	0	0	0	1	0	0	0	0	0	0	0	0	0	0	0	0	0	0	0	**1**
**17F**	0	0	0	0	0	0	0	0	0	0	0	0	0	0	0	0	1**	0	0	0	**1**
**18A**	0	0	0	0	0	0	1	0	0	0	0	0	0	0	0	0	0	0	0	0	**1**
**18F**	0	0	0	0	0	0	1	0	0	0	0	0	0	0	0	0	0	0	0	0	**1**
**19A**	0	0	0	0	0	0	0	5	0	0	0	0	0	0	0	0	0	0	0	0	**5**
**19F**	2	0	0	0	0	0	0	0	1	0	0	0	0	0	0	0	0	0	0	0	**3**
**20B**	0	0	0	0	0	0	0	0	0	1	0	0	0	0	0	0	0	0	0	0	**1**
**22A**	0	0	0	0	0	0	0	0	0	0	1	0	0	0	0	0	0	0	0	0	**1**
**22F**	0	0	0	0	0	0	0	0	0	0	0	0	0	1	0	0	0	0	0	0	**1**
**23F**	0	0	0	0	0	0	0	0	0	0	0	3	0	0	0	0	0	0	0	0	**3**
**3**	0	1	0	0	0	1^	0	1^	0	0	1	0	20^	1^	0	0	0	0	0	0	**25**
**35A/10B-like#**	0	0	0	0	0	0	0	0	0	0	0	0	1**	1	0	0	0	1**	0	0	**3**
**35B**	0	0	0	0	0	0	0	0	0	0	0	0	1	0	0	0	0	0	0	0	**1**
**4**	1^	0	0	0	0	0	0	0	0	0	0	0	0	6^	0	0	1^	1	0	0	**9**
**5**	0	0	0	0	0	0	0	0	0	1	0	0	0	0	0	0	0	0	0	0	**1**
**6A**	0	0	0	0	0	0	0	0	0	0	0	0	0	0	1	0	0	0	0	0	**1**
**6B**	0	0	0	0	0	0	0	0	0	0	0	0	0	0	0	1	1	0	0	0	**2**
**6D**	1^	0	0	0	0	0	0	0	0	0	0	1^	0	0	0	1	0	1	0	0	**4**
**7F**	0	0	0	0	0	0	0	0	0	0	0	0	0	0	0	0	4	0	0	0	**4**
**8**	1	0	0	0	0	0	0	0	0	0	0	0	0	0	0	0	0	16	0	0	**17**
**9L**	0	0	0	0	0	0	0	0	0	0	0	0	0	0	0	0	0	0	1	0	**1**
**9V**	0	0	0	0	0	0	0	0	0	0	0	0	0	0	0	0	0	0	0	1	**1**
**NT4a**	0	0	0	0	0	0	0	0	0	0	0	0	0	0	1	0	0	0	0	0	1
**Total**	**13**	**1**	**2**	**1**	**1**	**1**	**2**	**6**	**1**	**2**	**2**	**5**	**22**	**10**	**2**	**2**	**7**	**20**	**1**	**1**	**102**

*Two samples with 2nd serotype identified in NPS; **One sample with 2nd serotype identified in NPS; ^Detected as one of two or three serotypes on UAD assays
#New serotypes 33G (n=1)[11] or 33H (n=2)[12]

4. Supplementary Figures

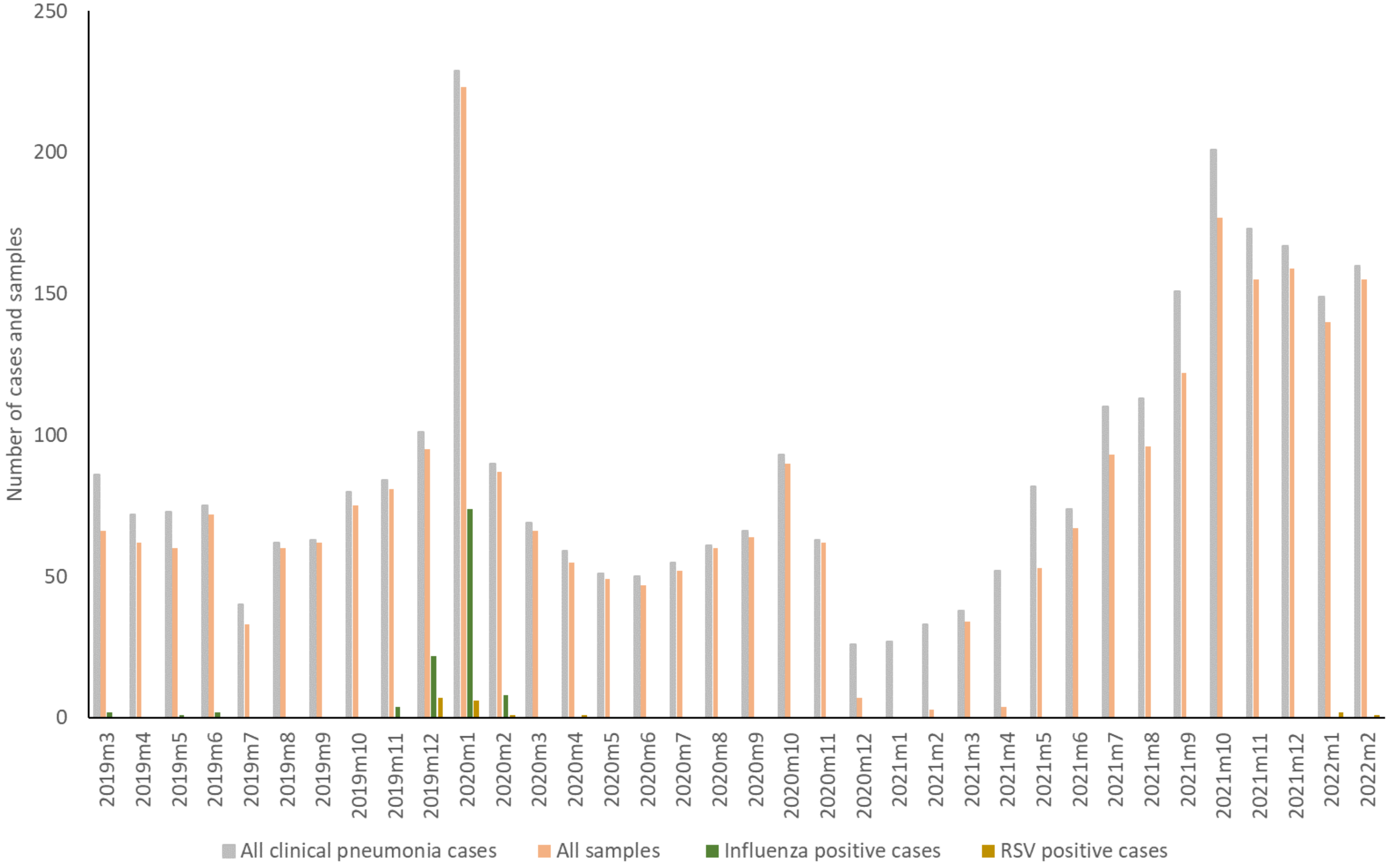

Supplementary Figure S1: Number of viral positive cases, detected in nasopharyngeal samples, in adults ≥18 years of age with clinical pneumonia, March 2019 to February 2022.

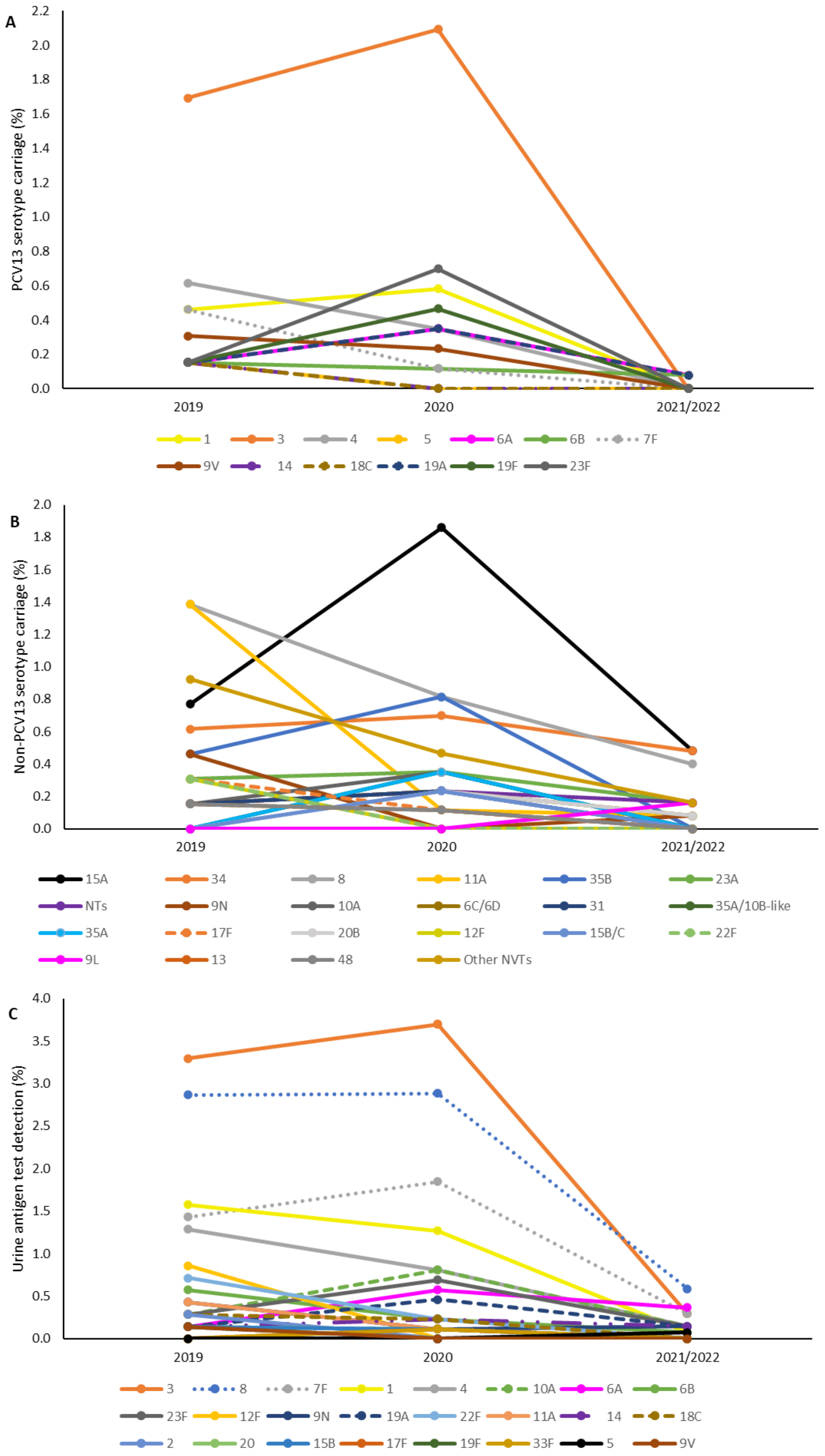

Supplementary Figure S2. Individual serotypes identified by study year in adults ≥18 years in Mongolia, March 2019 to February 2022

A. PCV13 serotype nasopharyngeal carriage, B. Non-PCV13 serotype nasopharyngeal carriage, C. Serotype specific urine antigen detection positive prevalence.

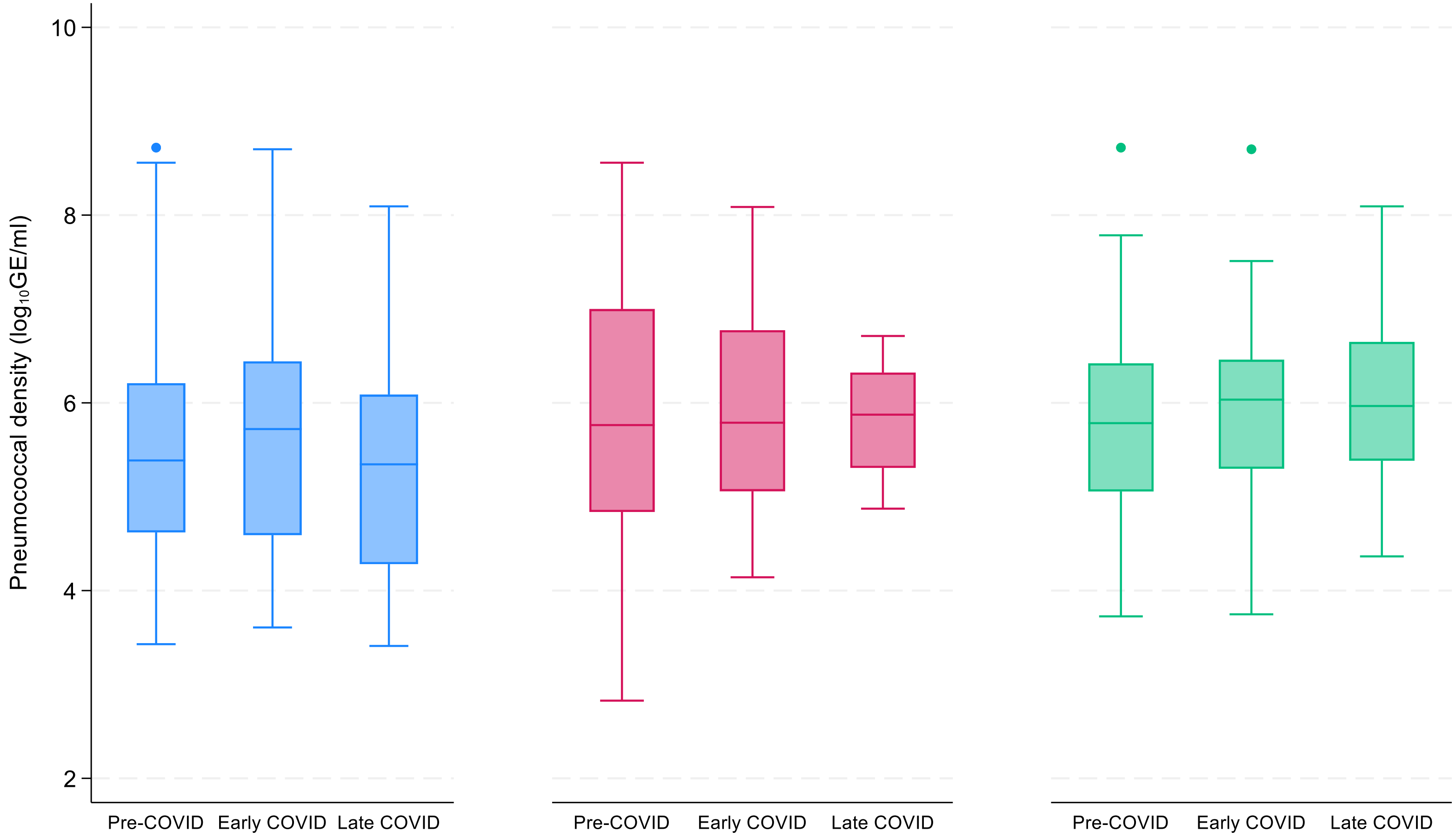

Supplementary Figure S3: Nasopharyngeal pneumococcal carriage density (log10 genome equivalents/ml) over the three COVID-19 periods in adult ≥18 years of age with clinical pneumonia who were positive for pneumococcal carriage. A. All pneumococci, B. PCV13 serotypes, C. Non-PCV13 serotypes
Boxes depict interquartile range (IQR) with a central line at the median, and whiskers extend 1.5 times IQR past the quartiles. Values outside whiskers plotted as individual points.

5. References

1. Satzke C, Dunne EM, Choummanivong M, et al. Pneumococcal carriage in vaccine-eligible children and unvaccinated infants in Lao PDR two years following the introduction of the 13-valent pneumococcal conjugate vaccine. *Vaccine* 2019;37(2):296-305. doi: https://doi.org/10.1016/j.vaccine.2018.10.077 [published Online First: 20181128]
2. Chen S, Zhou Y, Chen Y, et al. fastp: an ultra-fast all-in-one FASTQ preprocessor. *Bioinformatics* 2018;34(17):i884-i90. doi: 10.1093/bioinformatics/bty560
3. Langmead B, Salzberg SL. Fast gapped-read alignment with Bowtie 2. *Nature Methods* 2012;9(4):357-59. doi: 10.1038/nmeth.1923
4. Danecek P, Bonfield JK, Liddle J, et al. Twelve years of SAMtools and BCFtools. *GigaScience* 2021;10(2) doi: 10.1093/gigascience/giab008
5. Schneider VA, Graves-Lindsay T, Howe K, et al. Evaluation of GRCh38 and de novo haploid genome assemblies demonstrates the enduring quality of the reference assembly. *Genome Res* 2017;27(5):849-64. doi: 10.1101/gr.213611.116 [published Online First: 20170410]
6. Sheppard CL, Manna S, Groves N, et al. PneumoKITy: A fast, flexible, specific, and sensitive tool for *Streptococcus pneumoniae* serotype screening and mixed serotype detection from genome sequence data. *Microb Genom* 2022;8(12) doi: https://doi.org/10.1099/mgen.0.000904
7. Wood DE, Lu J, Langmead B. Improved metagenomic analysis with Kraken 2. *Genome Biology* 2019;20(1):257. doi: 10.1186/s13059-019-1891-0
8. Lu J, Breitwieser FP, Thielen P, et al. Bracken: estimating species abundance in metagenomics data. *PeerJ Computer Science 3:e104* 2017
9. Erkhembayar R, Dickinson E, Badarch D, et al. Early policy actions and emergency response to the COVID-19 pandemic in Mongolia: experiences and challenges. *Lancet Glob Health* 2020;8(9):e1234-e41. doi: https://doi.org/10.1016/S2214-109X(20)30295-3 [published Online First: 2020/07/28]
10. Byamba T, Tsilaajav T, Nyamsuren N, et al. Republic of Korea - World Bank Group Partnership on COVID-19 Preparedness and Response : Country Case Study - Mongolia (English) Washington, D.C.: World Bank Group; 2023 [Available from: http://documents.worldbank.org/curated/en/099110823205710240/P175398097720d0fb0aef006e07fedf91a2.
11. Manna S, Werren JP, Ortika BD, et al. *Streptococcus pneumoniae* serotype 33G: genetic, serological, and structural analysis of a new capsule type. *Microbiol Spectr* 2024;12(1):e0357923. doi: 10.1128/spectrum.03579-23 [published Online First: 20231207]
12. Manna S, Ortika BD, Werren JP, et al. Streptococcus pneumoniae serotype 33H: a novel serotype with frameshift mutations in the acetyltransferase gene wciG. *Pneumonia* 2025;17(1):7. doi: 10.1186/s41479-025-00162-2
